# How the congruence between public servants’ schemas and legal legitimacy affects top-down public policy implementation

**DOI:** 10.3389/fsoc.2025.1505494

**Published:** 2025-05-21

**Authors:** Jeferson Tadeu de Souza, Luciano Rossoni

**Affiliations:** Graduate Program in Administration, University of Brasília, Brasília, Brazil

**Keywords:** cultural schemas, regulatory legitimacy, public policy, legitimation, public servants, institutionalism, conformity

## Abstract

Why do some public policies mandated by the highest levels of government succeed, while others fail? This essay offers a partial answer by proposing that the effectiveness of a public policy depends on the congruence between the legal legitimacy of the law that establishes it, and the shared cultural schemas of the public servants tasked with its implementation. First, we contend that the effectiveness of top-down public policies enacted through laws and regulations relies on two key forms of validity: the legitimacy of the authority enacting the law—whether through constitutional or statutory frameworks—and the acceptability of the law’s content as something perceived as inevitable, fundamentally consensual, and recognized as a social obligation, reflecting the normative nature of the law. Second, we argue that top-down policies are more likely to be implemented effectively when their content aligns with the cultural meaning structures held by public servants. This alignment or congruence fosters a sense of ownership, increasing the likelihood of compliance with the law’s provisions. Finally, recognizing that a law’s legal validity and the propriety attributed to it by implementers may be only loosely coupled, we identify the conditions under which implementation is likely to result in conformity, customization, or ceremonial non-conformity. We conclude by discussing the practical and methodological implications of ensuring alignment between public servants’ cultural schemas and policy content, and we suggest empirical strategies to investigate this relationship.

## 1 Introduction

In the current landscape of public administration, the effectiveness of public policy implementation has gained increasing prominence (Pülzl and Treib, 2007; Robichau and Lynn, 2009). This growing relevance stems from the fact that modern democracies face complex challenges requiring government responses that are not only efficient but also legitimate (Parkinson, 2006). Indeed, citizens today expect more than just formal representation—they want their voices to be heard, and their demands addressed (Hanson, 2013). Within this context, both elected officials and bureaucratic institutions strive to develop public policies that respond to these expectations (West, 2004). However, even when such policies reflect public interests and rest on sound reasoning, their effective and successful implementation is not guaranteed (Lascoumes and Le Galès, 2007). These challenges are especially pronounced in the case of top-down policy implementation (Easterly, 2008), a common feature in modern democracies where governance operates across multiple institutional levels (Papadopoulos, 2003).

The literature on public policy identifies several obstacles to effective implementation. First, rationalist approaches tend to identify implementation barriers, such as resource scarcity and resistance to change, which hinder effective policy execution (Helbig et al., 2015). Moreover, within this same tradition, some authors emphasize the critical role of policy or state capacity, which refers to the need for governments and organizations to possess the skills and resources necessary for designing and implementing policies effectively (Gen and Wright, 2015). Second, pragmatic perspectives frame implementation challenges in terms of insufficient stakeholder engagement or inadequate socialization. For example, engaging relevant actors is seen as crucial, as it promotes buy-in and reduces resistance, thereby improving policy outcomes (Braun and Busuioc, 2020). Additionally, the choice between top-down and bottom-up approaches may significantly affect implementation effectiveness (Pülzl and Treib, 2007). Finally, institutionalist approaches from political science interpret implementation challenges as manifestations of institutional capacity, arguing that well-structured institutions are more capable of ensuring effective policy delivery (Olsen, 2008).

While these perspectives offer important insights, they often adopt an overly simplified view of the role of public agents in the implementation process. Approaches such as street-level bureaucracy (Lipsky, 1971, 1980; Maynard-Moody and Musheno, 2022) have highlighted how public servants’ daily practices and interpretations shape policy enactment. However, these approaches present two significant limitations. First, they lack a comprehensive theoretical framework for conceptualizing the *validity* of public policies (Tyler, 2006a,b; Tyler and Jackson, 2014; Walker, 2004)—that is, both the authority of the policymaker (Hadfield and Weingast, 2014) and the degree of legitimacy the policy holds in the eyes of those expected to comply with it (Dornbusch and Scott, 1975; Johnson et al., 2016; Tamanaha, 2011). Second, they do not adequately account for how public servants’ beliefs and interests are structured in ways that either facilitate or hinder implementation (Haack et al., 2021; Hunzaker and Valentino, 2019).

To address these gaps, in this essay, this essay argues that the effectiveness of public policy implementation depends on the congruence between the legal legitimacy of the policy and the shared meaning schemas held by public servants regarding its content. First, drawing on concepts from the sociology of law (Edelman and Suchman, 1999; Edelman and Talesh, 2011; Suchman and Edelman, 1996) and organizational institutionalism (Dornbusch and Scott, 1975; Scott, 2008; Haack et al., 2021), we contend that the success of top-down public policies enacted through legal instruments depends on two dimensions of validity: the legitimacy of the authority that enacts the law—whether grounded in constitutional or statutory provisions (Koskenniemi, 1997; Wallner, 2008)— and the normative acceptability of the law’s content, meaning its perception as inevitable, broadly consensual, and socially obligatory (Rosén, 2017; Rossoni, 2016; Walker, 2004). Second, drawing from the literature on cultural-cognitive sociology (D’Andrade, 1992; DiMaggio, 1997; Strauss and Quinn, 1997; Vaisey, 2009; Valentino, 2021; Zerubavel, 1997), we argue that top-down public policies are more likely to be successfully implemented when the regulation’s content resonates with the cultural schemas of public servants. Such alignment fosters a sense of ownership, increasing the probability of adherence. Finally, recognizing that the law’s validity and its perceived propriety may be only loosely coupled, we identify the conditions under which implementation is more likely to result in conformity, customization, or symbolic compliance (non-conformity).

To develop this argument, we organize the essay as follows: Section 2 reviews the main theoretical approaches to public policy implementation, framing implementation effectiveness as an organizational phenomenon. By aligning insights from the public policy literature with the lens of organizational institutionalism, we propose that policy implementation typically results in one of three outcomes: conformity, non-conformity, or customization. Section 3 argues that policies are more likely to be effectively implemented when they are legitimized both by the authority that enacts them and by the endorsement of the implementing agents—an idea rooted in the concept of legal legitimacy. Section 4 introduces the notion that policies require not only validity but also *propriety*, understood as the congruence between the policy content and public servants’ cultural schemas. Section 5 explores how varying combinations of legal validity and perceived propriety produce four distinct implementation outcomes, each associated with a specific strategic response. Illustrative examples are provided for each scenario. Finally, Section 6 addresses the methodological and practical implications of our framework for future research and policy practice.

## 2 Implementing top-down public policies: conformity, non-conformity, and customization

The concept of public policy (PP) can be defined in numerous ways depending on the analytical lens applied. Lowi (1964) offers a typological framework that classifies public policies into four categories—distributive, regulatory, redistributive, and constituent—based on their intended purposes. Lindblom (1965) in contrast, views public policy as a decision-making process aimed at addressing and resolving public problems, placing less emphasis on content and more on the dynamics of its development. For the purposes of this essay, we define public policy as a set of governmental actions designed to address and manage societal concerns (Fischer, 1980). These actions include directives, procedures, and regulations that structure the relationship between the State and the social actors who are the intended beneficiaries of public resources and services (Torrens, 2013). In practice, public policies are implemented through programs, funding mechanisms, administrative structures, and legislation that collectively reflect the priorities and characteristics of a given political regime. Public policy is often conceptualized as a cyclical process composed of successive stages, including agenda-setting, formulation of alternatives, decision-making, implementation, and evaluation (Secchi et al., 2020).

Although the formulation and evaluation stages are important for overall policy success (Sidney, 2007), our focus in this essay is on the implementation phase. Public policy implementation refers to the set of actions carried out by administrators, managers, and frontline public servants following the formal enactment of a policy by elected officials (Pan and Zhang, 2022; Robichau and Lynn, 2009; Smith, 1973). This is not to say that earlier stages are inconsequential—on the contrary, legal frameworks play a crucial legitimating role and significantly influence implementation dynamics (Edelman and Suchman, 1999; Sabatier and Mazmanian, 1980; Suchman and Edelman, 1996). Our analysis, however, begins with the point at which policy regulations are defined by higher authorities and proceeds to examine how these are interpreted and adopted by implementing agents, that is, public servants.

The dominant approaches to understanding policy implementation can be grouped into three categories: top-down, bottom-up, and hybrid. Each presents distinct implications for how policy guidelines are developed and executed (Pülzl and Treib, 2007). The top-down approach emphasizes a hierarchical model, wherein decisions are made at the central government level and carried out by subordinate actors (Sabatier and Mazmanian, 1980; Sabatier, 1986). This model assumes that policy success hinges on the clarity of objectives and the extent of control exerted over implementers (Pressman and Wildavsky, 1973). Foundational texts in this tradition adopt a prescriptive stance (Pülzl and Treib, 2007), viewing implementation as a rule-bound process requiring strict adherence to centrally defined mandates, with limited scope for interpretation (Lima and D’Ascenzi, 2013).

In contrast, the bottom-up approach challenges the hierarchical view by emphasizing the role of local-level implementers as de facto policymakers (Lipsky, 1980). It emphasizes how policy is shaped by contextual factors and the everyday interactions between street-level bureaucrats and the citizens they serve (Elmore, 1980). Furthermore, a central feature of this approach is its recognition of the broad discretionary power exercised by frontline public servants, which allows them to significantly influence implementation outcomes (DeLeon and DeLeon, 2002). This discretionary authority, in turn, is grounded in their ability to interpret, adapt, or even resist official directives in response to situational realities (Lipsky, 1971).

Top-down and bottom-up approaches differ not only in their levels of analysis, with the former emphasizing centralized control and the latter focusing on local autonomy. The top-down model emphasizes hierarchical coordination, clear lines of authority, and a more elitist view of representative democracy. The bottom-up model, by contrast, favors networked policy processes, local problem-solving, and a more participatory conception of democratic governance (Pülzl and Treib, 2007).

Given the various tensions between the top-down and bottom-up —and in response to the growing debate among their respective proponents (Matland, 1995), some scholars proposed a hybrid approach (Elmore, 1985; Ripley and Franklin, 1982; Sabatier, 1986). This perspective seeks to reconcile elements of both approaches by integrating centralized policy directives with local-level adaptations informed by implementers’ experience, allowing for enhanced adaptability without sacrificing policy coherence. The hybrid approach also facilitates the incorporation of diverse theoretical lenses and encourages the use of mixed methodologies—such as longitudinal case studies and network analysis—resulting in more robust, context-sensitive evaluations. It improves the practical utility of scientific findings by considering both political feasibility and organizational adaptability, thereby supporting more effective decision-making by policymakers and public managers (Pülzl and Treib, 2007).

Together, these three perspectives provide a comprehensive framework for analyzing public policy implementation, balancing the rigidity of legal structures with the complexity and unpredictability of real-world contexts. Accordingly, while this essay primarily focuses on top-down policy processes, it does so with an awareness of this broader analytical landscape. It is important to highlight, however, that most traditional implementation approaches are grounded in an objectivist ontological stance (Pülzl and Treib, 2007), which contrasts with the cultural-cognitive assumptions that guide our analysis. These assumptions bring us closer to interpretive perspectives on implementation. As a result, we emphasize the role of values, beliefs, and shared meaning schemas among implementing agents—an orientation that leads us to adopt organizational institutionalism as our theoretical framework (Scott, 2008; Suchman and Edelman, 1996).

Applying the lens of organizational institutionalism to public policy implementation, we identify three primary outcomes: conformity, non-conformity, and customization. Similar to organizations, conformity occurs when public organizations adhere to legally legitimized assumptions embedded in policy frameworks and justify their actions in accordance with institutional norms (Amirkhanyan et al., 2017; DiMaggio and Powell, 1983; Edelman and Talesh, 2011) and stakeholder demands. In contrast, non-conformity arises when organizations resist institutional pressures, whether due to resource constraints, strategic or normative conflicts, or a desire for innovation (Oliver, 1991). In the context of policy implementation, such resistance may manifest as ceremonial compliance, where formal adherence masks underlying noncompliance (Silva and Rossoni, 2024).

By comparison, customization occurs when public organizations adhere to regulatory principles while adapting them to better suit internal operational needs or specific contextual demands (Raffaelli and Glynn, 2014; Scott, 2008; Westphal et al., 1997). Greenwood et al. (2017) emphasize that customization reflects an organization’s capacity to navigate and balance external institutional expectations with internal practical realities—a dynamic equally relevant to public sector institutions. While conformity contributes to institutional legitimacy and stability, both non-conformity and customization provide avenues for strategic flexibility and innovation.

## 3 Legal legitimacy and public policy implementation

Legitimacy refers to a widespread belief that the actions of entities, organizations, governments, and policies (including public policies) are appropriate and consistent with a socially constructed system of norms, values, beliefs, and definitions (Meyer and Scott, 1983; Suchman, 1995). This understanding draws on the Weberian notion that a social order is only considered legitimate if it is guided by identifiable and accepted maxims (Scott, 2008). Even in cases where individuals do not share identical values and beliefs, their behavior is often oriented by this overarching order (Johnson et al., 2006). As individuals perceive that others endorse this social framework, it begins to appear as an objective social fact—something natural and inevitable—serving as a model for appropriate conduct (Walker, 2004). Indeed, as Berger and Luckmann (2008, pp. 128, 129), legitimacy “not only tells the individual why they should perform one action rather than another; it tells them why things are the way they are.”

Legal legitimacy, in turn, can be understood as the perceived obligation to obey the law (Halliday, 2023). In this sense, a law is considered valid if individuals feel morally or socially compelled to follow its provisions, thereby conferring legitimacy upon the legal order itself. This implies a normative alignment between society and the legal structures established by the state: when individuals recognize state power as legitimate, they are more inclined to comply with it (Beetham, 2013). This definition is both a felt duty to obey and an affective commitment to legal authorities (Tyler, 2006b; Tyler and Jackson, 2014), thus extending the idea of legal legitimacy beyond the procedural legitimacy of the legal system itself.

This understanding of legal legitimacy—as a behavioral expectation whereby individuals follow the law not only out of obligation but also because they believe in its underlying validity, even when it is imperfect—corresponds to what Dornbusch and Scott (1975) and Johnson et al. (2016) refer to as validity by endorsement. Rooted in Weberian theory, this form of legitimacy is based on the belief that acceptance of the law is inevitable due to a shared social consensus (Johnson et al., 2006; Walker, 2004; Zelditch, 2001). From this perspective, the law is seen as “a normative system that resides in the minds of the citizens of a society” (Carothers, 2006, p. 20); that is, compliance is not necessarily the result of the law’s content but rather of its embeddedness in the collective social order (Tamanaha, 2011).

Viewing law and regulations as contingent upon normative validation introduces an important dimension to the concept of legal legitimacy (Halliday, 2023). Yet, another source of legitimacy arises from the belief that legal authorities are entitled to determine appropriate behavior and conduct (Hadfield and Weingast, 2014). Indeed, this form of validity by authorization rests on the perceived legitimacy of those who enact the law (Walker et al., 1986). This distinction allows us to differentiate between two sources of public policy legitimacy: *authorization* and *endorsement*. Legitimacy by authorization stems from the legal authority of those who issue the policy—whether derived from constitutional provisions or formally enacted statutes (Dornbusch and Scott, 1975), such as the Constitution. Legitimacy by endorsement, in contrast, emerges when individuals believe that others will comply with the policy, either because it is widely observed or perceived as inevitable—thereby reinforcing the perceived rationality and validity of the policy’s content and foundation (Weinberger, 1999).

An indication of validity by authorization is the belief that legal authorities have the rightful power to prescribe behavior (Tyler and Jackson, 2014). In this case, individuals comply—or choose not to comply—with an authority figure based on whether that figure’s “right to power” is viewed as legitimate within prevailing social norms (Gordon et al., 2009). In this sense, authority is maintained through a structure of domination supported by socially established norms that legitimize the coercive capacity of the state (Gordon et al., 2009).

From a structural standpoint, the rule of law is typically organized around three foundational institutions (Hadfield and Weingast, 2014): (1) accessible and stable laws; (2) a qualified and independent judiciary; (3) an effective mechanism for law enforcement and public order (Kleinfeld, 2006). This framework is especially relevant in the context of public policy, as public servants function as the “effective force” responsible for implementing such policies. Their actions must be informed both by their interpretation of the law and by the jurisprudential guidance provided by the judiciary.

In addition, it is essential to consider that democratic legal systems operate within a hierarchical structure of legal norms (Wihlborg, 2005), which refers to the ranking of legal “norms” and principles within a legal system, according to their legal authority or binding force (Shelton, 2006). Accordingly, when a public policy is enacted at higher levels within the legal hierarchy, it is presumed to carry greater legal validity (Kelling, 2024) and, therefore, more likely to be perceived as legitimate.

Most legal systems organize this hierarchy into three primary levels (Kelsen, 1945; Koskenniemi, 1997): (1) at the highest level, the constitution defines the fundamental values of a society and supersedes all other laws (Shelton, 2006); (2) beneath the constitution are ordinary laws and legal codes, which must conform to constitutional principles and are themselves internally ordered, with codes typically carrying greater legal weight (Butt and Lindsey, 2018); and (3) at the lowest level are administrative regulations, which, while not always requiring legislative approval, must comply with higher legal norms and serve primarily to operationalize policy through implementation guidelines (Shelton, 2006).

It is particularly important to note that public policy implementation is most often regulated at the lowest tier of this hierarchy (Jagers et al., 2020). Although high-level laws and regulations establish the general principles and boundaries of public policy (Streeck and Thelen, 2005; Theodoulou and Cahn, 1995), they tend to be broad and sometimes contradictory or ambiguous, offering limited practical guidance to public servants (Edelman and Talesh, 2011). As a result, it frequently falls to implementers—especially street-level bureaucrats—to interpret and adapt these laws to practical, real-world circumstances (Kelling, 2024; Von Bogdandy et al., 2008; Yakovleva et al., 2022).

Thus, the legitimacy and effectiveness of public policy depend on its alignment with the expectations of both stakeholders and the broader public (Morhunov et al., 2023; Wallner, 2008). Achieving such alignment is significantly more feasible when the policy formulation process incorporates elements perceived as essential and non-negotiable by society—this corresponds to *validity by endorsement*. At the same time, legitimacy is also shaped by the legal status of the regulation and the institutional authority of its issuer—what we refer to as *validity by authorization*. Taken together, these two forms of legal validation form the basis of a public policy’s legitimacy, and, therefore, we propose that:

*Proposition 1: The greater the legitimacy of the law or regulation that establishes a public policy—both in terms of the authority of the enacting body and the normative acceptance of its content—the greater the likelihood of conformity with its intended purpose.*

## 4 Assigning propriety to public policies: public servants’ cultural schemas

Although a law or regulation establishing a public policy may be legitimate in terms of its validity and the authority from which it originates (Tyler, 2006a), it is not uncommon for such policies to be poorly implemented—even when they carry full legal legitimacy. Theories of legitimacy suggest that while there may be a general consensus on adherence to social obligations, these obligations are not always internalized by individuals. When internalization fails, conformity can be weakened (Dornbusch and Scott, 1975; Haack et al., 2021; Johnson et al., 2006; Rossoni, 2016; Walker et al., 1986; Walker, 2004; Zelditch, 2001).

To better understand such deviations from what is considered legitimate, Dornbusch and Scott (1975) drawing on classical Weberian thought, argue that legitimacy requires more than just *validity*—it must also exhibit *propriety* in the eyes of those evaluating the legitimate object (e.g., a law or regulation enacting a given public policy). Propriety refers to an actor’s belief that the norms and procedures of a given rule or institution constitute desirable and appropriate standards of behavior (Dornbusch and Scott, 1975; Johnson, 2004; Walker, 2004; Zelditch, 2001). In other words, propriety captures whether the essence, attributes, or actions associated with the object are appropriate within its social context (Haack et al., 2021). Thus, whereas validity reflects a sense of duty or obligation shaped by shared expectations, propriety concerns whether individuals perceive the object as normatively justified or worthy of support (Dornbusch and Scott, 1975; Rossoni, 2016).

The degree of propriety attributed to a legitimate object can have substantial behavioral consequences. When something is both valid and perceived as proper (i.e., possessing propriety), it tends to serve as a compelling model for action (Walker, 2004). Conversely, even a valid policy may be viewed as illegitimate if it is seen as misaligned with prevailing expectations (Haack et al., 2021). This becomes particularly evident in situations of severe norm violation—such as ethical scandals or misconduct (Mac Lean and Behnam, 2010), when individuals, disillusioned with institutional behavior, begin to question the legitimacy of the norms and authorities that were previously accepted.

In the realm of public policy implementation, public servants constitute the primary audience that confers propriety on a legally valid law or regulation. Therefore, the concept of propriety emphasizes the importance of frontline officials’ acceptance of the principles embedded in a regulated public policy. It also highlights the critical role of individual-level legitimacy in the broader legitimation process—an aspect often neglected in organizational institutionalism (Haack et al., 2021). However, while the literature on organizational legitimacy acknowledges the relevance of audience-based propriety, it typically treats this construct as an independent variable without fully exploring the reasons why individuals consider a policy or regulation appropriate. To address this gap, we propose that propriety can be explained through the *congruence* between the content of the legitimate object (i.e., the policy regulation) and the *shared meaning schemas* of public servants.

Because the propriety of a legitimate object is shaped by beliefs that are socially constructed and culturally embedded, we conceptualize propriety in terms of *cultural schemas*. As DiMaggio (1997, p. 269) defines them, cultural schemas are “knowledge structures that represent objects or events and provide default assumptions about their characteristics, relationships, and entailments under conditions of incomplete information.” These schemas consist of networks of associated ideas formed through repeated experiences. They are essential for understanding automatic cognition (Boutyline, 2017; Goldberg, 2011; Hunzaker and Valentino, 2019) as they allow individuals to interpret information and guide action without the need for conscious deliberation. Yet, schemas shape how people perceive the world and influence their responses to varying situations (Miles et al., 2019). Cultural schemas may be innate or acquired through lived experience and acculturation (Zerubavel, 1997), organizing knowledge about reality (Strauss and Quinn, 1997), and informing how individuals perceive and interpret cultural practices (Boutyline and Soter, 2021; DiMaggio, 1997).

Although cultural schemas are socially shared, they are embodied and operate at the individual level (Boutyline and Soter, 2021; Hunzaker and Valentino, 2019). They function automatically, without the need for conscious deliberation, organizing, and interpreting information based on internalized cultural norms and experiences (Boutyline and Soter, 2021; Hunzaker and Valentino, 2019). Furthermore, they are context-sensitive and activated in specific situations to guide appropriate behavior in line with socially recognized expectations (Boutyline and Soter, 2021; DiMaggio, 1997; Hunzaker and Valentino, 2019).

In light of these considerations, it is important to note, however, that not all schemas are cultural in nature (Boutyline and Soter, 2021; Strauss and Quinn, 1997). To qualify as cultural, a schema must meet two key criteria. First, it must be representational—it should convey information about the world in a way that shapes automatic cognition, transmitting meanings through assumptions, categories, and normative scripts that structure perception and action (Cerulo et al., 2021). Second, it must be widely shared within a social group, rather than being merely personal or idiosyncratic (Patterson, 2014). Although cultural schemas are collectively held, they are internally represented and embodied by individuals (Patterson, 2014), meaning that identifying them requires careful analytical methods capable of capturing how personal representations scale up to collective meaning structures (Hunzaker and Valentino, 2019; Homan et al., 2017; Valentino, 2021).

To identify shared meaning structures, we adopt a “culture as schematic similarity” perspective (Martin, 2002; Rossoni et al., 2021), which holds that content is cultural insofar as it reflects consistent cognitive patterns among individuals within a defined social group (Hunzaker and Valentino, 2019; Patterson, 2014; Strauss and Quinn, 1997; Zerubavel, 1997). This shared similarity in schemata—shaped through common experiences and processes of cultural transmission—is paramount. From this standpoint, culture manifests at various levels, from familial and organizational to professional and national spheres. Each “culture” comprises a system of elements (e.g., beliefs, values, attitudes, meanings, knowledge, schemas, artifacts, and practices) that members of a group interpret and organize in broadly similar ways (Hunzaker and Valentino, 2019).

D’Andrade (1992) emphasized that cultural schemas capture both the procedural and interpretive aspects of motivation by facilitating action that is guided by culturally derived meanings. According to this author, schemas have motivational force because they help individuals make sense of and respond to situations. He also argues that schemas vary in specificity and autonomy, making it possible to understand motivations as part of a broader interpretive system. Moreover, D’Andrade (1992) suggests that schemas can possess “directive force,” exerting a sense of moral obligation that reinforces the link between cultural meaning and motivated behavior.

Given the motivational role of cultural schemas, we argue that the effect of legal legitimacy on implementation is mediated by the propriety attributed to the policy or regulation. This mediation process can only be fully understood by analyzing the cultural schemas through which public servants interpret and operationalize laws as actionable guidelines. In this regard, schema theory offers a valuable analytical framework for investigating the specific content of public servants’ beliefs about the legitimacy of public policy (Homan et al., 2017).

Accordingly, we define public servants’ cultural schemas as shared cognitive structures that shape how they interpret and enact public service norms, policies, and institutional routines. Drawing on cultural schema theory (D’Andrade, 1992; DiMaggio, 1997; Strauss and Quinn, 1997; Zerubavel, 1997), we understand these schemas as associative networks of concepts internalized through bureaucratic practice, organizational socialization, and cultural learning within state institutions.

These schemas are activated in specific contexts (Boutyline and Soter, 2021; Hunzaker and Valentino, 2019), influencing how public servants perceive the legitimacy of regulations and guidelines, interpret their responsibilities, and make decisions in the course of their duties. Like other cultural schemas, they are collectively held within a social group—in this case, the bureaucratic field—and reflect similar cognitive patterns that shape how norms are understood, interpreted, and applied in public administration.

Although cultural schemas are collectively shaped by institutional contexts, they operate at the individual level, guiding automatic cognition and behavior without requiring conscious deliberation (Goldberg, 2011; Patterson, 2014). Moreover, varies depending on the specificity of the institution, the function performed, and interactions with different social and political groups, making them central to the dynamics of public policy implementation (Homan et al., 2017). We therefore assume that the various networks of meaning composing these cognitive structures correspond to distinct cultural schemas (Boutyline, 2017; Goldberg, 2011); that is, diverse ways of understanding what a public policy is and how it should function.

### 4.1 Congruence between legal regulation content and public servants’ schemas

Differences among public servants’ cultural schemas can be identified by comparing networks of meaning associated with concepts and beliefs that hold supra-individual relevance (Goldberg, 2011). The literature suggests that distinct groups exhibit divergent logics in both the organization of knowledge related to public policies (Goldberg, 2011; Valentino, 2021; Willekens and Daenekindt, 2022) and in the evaluation of individual policy elements (Patterson, 2014). Thus, each group or category of public servants characterized by a particular schema also reflects distinct logic—cognitive structures that are shared, internalized, and evaluative in nature (Valentino, 2021). While schemas capture relationships among concepts (e.g., how public servants classify types of policy), cultural logic captures evaluative judgments and meanings assigned to these concepts and their place in the social world (Cerulo et al., 2021; Hunzaker and Valentino, 2019; Rossoni et al., 2021; Valentino, 2021).

This aspect of judgment or evaluation in public policy relates to how the content of a regulation that establishes a policy is assessed by the public servant. Given that both the policy content and public servants’ schemas are multifaceted, the question of when a policy is legitimately appropriate does not depend solely on group consensus or direct alignment with its content (Dressler et al., 2019; Dressler, 2020), but rather on the degree of congruence between the law and the various elements that compose public servants’ cultural schemas.

The concept of congruence refers to the degree of proximity or distance between the beliefs and attitudes of public servants and the content of the regulation that enacts the policy. This idea resembles measures of ideological congruence, which examine the alignment between citizens’ interests and those of their representatives (Golder and Stramski, 2010), as well as the notion of congruence with social categories, which reflects the consistency of individual beliefs with group identities (Ajzen and Sexton, 1999). However, it aligns more closely with the notion of value congruence, as explored by Lu et al. (2024), who define cultural fit as the match between an individual’s values and those prevailing within a given entity or organization.

Despite these conceptual similarities, the definition of congruence between public servants’ cultural schemas and the content of legal regulations involves some specific characteristics: (1) it considers how the belief system of the public servant, an intrinsic factor, relates to the content of the law, an extrinsic or exogenous factor; (2) this belief system is multifaceted, composed of a set of beliefs and attitudes that shape how public servants evaluate the law; (3) it is also relational, meaning that the connections between different beliefs and attitudes contribute to the formation of intention; and (4) it is socially shared, such that analyzing similarities across networks of beliefs and attitudes among public servants enables the identification of distinct cultural schemas.

Considering these four properties, we propose that the congruence between public servants’ cultural schemas and the content of legal regulations corresponds to the valence attributed to each belief and attitude internalized by public servants regarding the normative elements of the law—ultimately influencing how policies are interpreted, evaluated, and implemented. In line with this definition, we argue that top-down public policies have greater potential for successful implementation when their content aligns with the shared meaning schemas of public servants. This congruence fosters a sense of ownership (Walker, 2004), thereby increasing the likelihood that the law’s provisions will be followed. Conversely, when this congruence is lacking, it can lead to resistance and hinder effective policy implementation (Bruni, 2021; Hunzaker and Valentino, 2019). Accordingly, we propose:

*Proposition 2: The greater the congruence between the content of a law or regulation that enacts a public policy and the cultural schemas of public servants, the higher the perceived propriety, thereby increasing conformity with its intended purpose during implementation.*

## 5 Interacting legal legitimacy and congruence of public servants’ schemas: effects on public policy implementation

The arguments presented thus far suggest that the implementation of public policy aligns with its intended purpose through two interrelated mechanisms: *validity*, ensured by the legitimacy of the law or regulation that enacts it (Proposition 1), and *propriety*, derived from the congruence between its content and public servants’ cultural schemas (Proposition 2). However, since validity and propriety represent distinct dimensions of the legitimation process (Haack et al., 2021; Johnson et al., 2006, 2016; Walker, 2004), it is possible for a policy to exhibit one without the other—that is, a policy may possess legal validity but lack propriety, or conversely, be perceived as proper but lack legal legitimacy. In such cases, what expectations should we have regarding compliance with the policy? And how might public servants respond to different combinations of these two dimensions?

To answer these questions, we adopt the premise that legitimacy functions as a motivator for action (Vaisey, 2009). On one hand, validity facilitates the internalization of regulation as a general disposition toward action. On the other hand, propriety, stemming from the congruence between a regulation’s content and cultural schemas, is what activates or enacts this disposition (Cerulo et al., 2021; Miles et al., 2019; Walker, 2004). In this sense, a valid regulation—embedded within a rationalized framework of public service—manifests as a “duty” (Giddens, 1984), being closely linked to a perceived legitimate purpose (Abrutyn and Lizardo, 2024). Thus, the greater the legitimacy of the law that establishes a public policy, the greater its potential to motivate public servants toward compliance with its intended goals.

However, to fully understand the motivational effect of legitimacy, it is not enough to treat legal validity as a binary perception of duty. Regardless of whether the law is seen as obligatory, a public policy may still be perceived as more or less *desirable* (Edelman and Suchman, 1999; Kelling, 2024). Public servants whose schemas align with the normative spirit of a policy are likely to find satisfaction in its implementation, which in turn motivates them to act (Abrutyn and Lizardo, 2024; D’Andrade, 1992). Conversely, when the content of a policy diverges from their internalized schemas, its implementation becomes less desirable, diminishing their motivation to carry it out (Edelman and Talesh, 2011; Olsen, 2008).

Based on this reasoning, we argue that the effectiveness of public policy varies depending on how *validity* (low or high) and *propriety* (low or high) interact. The combination of these factors leads to different implementation outcomes: conformity, customization, or non-conformity (Silva and Rossoni, 2024; Westphal et al., 1997). Furthermore, we hypothesize that each scenario is associated with a distinct strategic response: commitment, persuasion, manipulation, avoidance, or defiance (Elbers and Arts, 2011; Nguyen et al., 2023; Oliver, 1991).

When contradictions arise between validity and propriety, we distinguish whether they stem from conflicts related to the means or the goals of policy implementation (Oliver, 1991; Pache and Santos, 2010). In this context, we define public policy institutional demands as the pressures for conformity exerted by institutional referents on organizations operating within a given field (Pache and Santos, 2010). We refer to conflicting institutional demands as tensions that emerge from incompatible expectations across different components of the governmental apparatus (Silva and Rossoni, 2024).

**Figure 1:**
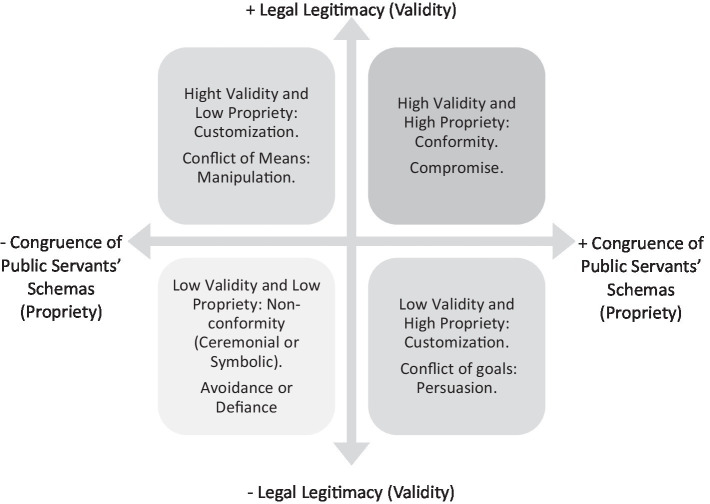
Interacting legal legitimacy and congruence of public servants’ schemas.

As an analytical effort to systematically represent these relationships between propriety and validity, we theorize these relationships in a matrix summarized in Figure 1. The vertical axis represents validity, attributed to the legal legitimacy of the regulation that establishes a public policy, ranging from low to high. The horizontal axis reflects the degree of congruence between the content of that regulation and the cultural schemas of public servants. The combination of these two dimensions yields four typical scenarios, each associated with varying degrees of implementation effectiveness and corresponding strategic responses. While we describe these quadrants as distinct ideal types, we emphasize that public policy legitimacy should be understood as a continuum, shaped by varying degrees of both validity and propriety.

### 5.1 High validity and high propriety: conformity

In situations where the law or regulation that establishes a public policy is valid—either because it is endorsed by those affected or because of the authority of its promulgating entity—and simultaneously aligns with the beliefs and expectations of the public servants responsible for its implementation, a high degree of conformity with its provisions is anticipated. Consequently, the most likely strategic response from public servants is one of compromise (Oliver, 1991). This occurs not merely out of compliance with the law but because its content is perceived as legitimate, as it aligns with their internalized cognitive frameworks.

This type of response is highly desirable for any government committed to the effective implementation of public policy and is often taken for granted in implementation manuals and policy design models (Pülzl and Treib, 2007). However, such ideal conditions tend to be more the exception than the rule in top-down policy implementation, which explains their relative scarcity in the empirical literature. When these cases do arise, frequent questions include why policies perceived as both valid and appropriate took so long to be implemented, or how they evolved into such a state of alignment.

One illustrative example is provided by Hannigan and Kueneman (1977), who analyzed the changing legitimacy of Canada’s Emergency Measures Organization, initially created to respond to nuclear threats during the Cold War. In its early years, the organization operated with uncontested legitimacy, as nuclear preparedness was viewed as a strategic necessity. However, as the perceived atomic threat subsided, the organization’s legitimacy eroded, leading to budget cuts and a decline in political support. The legitimacy crisis was eventually resolved when the agency reoriented its mission toward natural disaster management, thereby realigning its objectives with emerging social demands and restoring congruence with both public expectations and bureaucratic priorities.

### 5.2 Low validity and high propriety: customization focusing on conflict of goals

In cases where a law or regulation lacks validity—whether due to procedural flaws in its enactment or the limited legitimacy of the issuing authority within the democratic hierarchy—but nevertheless aligns with the expectations and beliefs of public servants regarding its operationalization, tensions tend to emerge around the goals, purposes, or normative assumptions that underpin the policy. In such contexts, uncertainty may arise concerning how the policy is justified to its target audience, as well as the degree of resistance encountered during implementation.

This situation often leads public servants to customize the implementation process in an effort to minimize conflicts surrounding the policy’s goals. Given that there is typically less disagreement over the means by which the policy’s objectives might be achieved, the most likely strategic response is persuasion (Elbers and Arts, 2011; Nguyen et al., 2023). This tactic involves offering compelling arguments and rationales—particularly effective in settings where interpersonal trust and direct engagement exist (Elbers and Arts, 2011). Persuasion is considered less coercive than control and more relational than negotiation (Oliver, 1991), often materializing through explanatory dialogue or public communication campaigns (Nguyen et al., 2023).

Numerous examples of this type of scenario appear in the literature. For instance, Jackson et al. (2023) examine how certain regulations continue to be enforced even when their validity is questioned or when the authorities behind them lack strong political support. The authors cite cases involving environmental standards and workplace safety regulations that were implemented without widespread democratic consultation or public consensus, resulting in legitimacy challenges. Nevertheless, such policies remained in force because inspectors and enforcement agents were already embedded in established bureaucratic routines and technical protocols. In this sense, the congruence between the policy content and bureaucratic practice enabled continued enforcement despite weak formal legitimacy.

Another pertinent example comes from Lim and Sloan (2016), who investigated the factors influencing the willingness of 553 senior law enforcement officers in Texas to report misconduct among their peers. Although whistleblower protections may lack clear legal backing or may face resistance due to institutional inertia, the study found that some officers were nevertheless willing to act because the content of these policies resonated with their professional values—particularly a shared commitment to institutional integrity. This case exemplifies a scenario in which the law may lack full formal validity but is perceived as proper by its implementers, thereby facilitating its enforcement.

### 5.3 Low validity and low propriety: non-conformity, ceremonial or symbolic

Some public policies are instituted without adequate legal or normative legitimacy (Gordon et al., 2009). Many are driven by media salience (Spirig, 2023; Tan and Weaver, 2009), while others stem from arbitrary decisions made by government officials (Teter, 2012). In addition to lacking legal legitimacy, these policies often also lack propriety—that is, they fail to resonate with the beliefs or expectations of the public servants responsible for their implementation. Under such circumstances, the likelihood of non-conformity is high (Westphal et al., 1997), with implementation efforts reduced to ceremonial or symbolic acts rather than substantive compliance.

As a result, one of the most common strategic responses in this context is avoidance, wherein public servants evade compliance with the requirements imposed by regulatory authorities (Pache and Santos, 2010). Tactics of avoidance may include concealing non-compliance, insulating the organization from external scrutiny, or circumventing institutional norms and expectations (Oliver, 1991).

Another possible, though less frequent, response is defiance, which involves overt resistance to policy directives (Oliver, 1991). Defiance tends to be riskier due to its confrontational nature, particularly when directed at governing authorities. Oliver (1991) outlines three core tactics of defiance: (a) ignoring or dismissing institutional rules, especially when enforcement mechanisms are weak; (b) openly challenging rules to mobilize public opinion or provoke legal reform; and (c) directly opposing institutions or actors to pressure change through coercive means.

Examples of non-conformity are well documented in the literature. Coombs (1992) analyzes the case of the Task Force on Food Assistance, established by the Reagan administration in 1983. Although hunger was recognized as a legitimate concern, the task force faced widespread resistance due to the perceived lack of expertise among its leaders. Additionally, its central proposal—to replace federal food assistance programs with block grants to states—was met with skepticism and ultimately rejected by both legislators and policy experts. The proposal had already failed in prior attempts, and frontline implementers viewed it as impractical. Congress ultimately disregarded the task force’s recommendations and passed legislation to maintain federal support.

Brønstad and Berg (2011) examined the low compliance with the 3Rs principles—replacement, reduction, and refinement—in animal research. They found that many researchers showed low adherence to ethical principles governing animal experimentation because these guidelines often clashed with prevailing norms and practices within scientific communities. The authors argue that deeply rooted cultural frameworks can precede and override formal legal structures, creating a persistent gap between regulatory requirements and actual behavior in the field.

### 5.4 High validity and low propriety: customization focusing on conflict of means

Finally, the most frequently discussed scenario in the literature involves public policies that possess high validity—they are properly enacted and grounded in legal authority—but lack propriety, due to misalignment with the cultural schemas of public servants. Despite being rooted in legitimate purposes, such policies often encounter resistance from implementers when aspects of the policy contradict their beliefs or fail to meet standards they consider meaningful or necessary (Olsen, 2008). Moreover, given that every policy is shaped by uncertainty and causal ambiguity (Cairney et al., 2016), public servants’ schemas play a vital role in shaping how policies are judged and enacted (DiMaggio, 1997).

Due to the need to adjust expectations and interests in light of the low propriety of public policy, public servants perceive customization as necessary for the policy to be effective (Silva and Rossoni, 2024). Since demands for customization are linked to public servants’ willingness to accept the policy—and, consequently, to mobilize in order to implement it—conflicts emerge over the means through which this implementation should occur (Pache and Santos, 2010). As incongruence inherently heightens resistance and disagreement, the most common strategic response among powerful actors is manipulation (Nguyen et al., 2023). Oliver (1991) defines manipulation as an intentional and strategic attempt to co-opt, control, or steer individuals or groups. It is the most active form of response to institutional pressures, as it seeks to directly influence or alter institutional expectations or their sources.

There are numerous cases of valid but improper policies in the literature. Andrews (2008) discusses reforms based on the New Public Management (NPM) model, which illustrate this contradiction: while governments legitimize and adopt such reforms to improve public sector efficiency, public servants often reject them because they conflict with their professional values and practices. In countries with prominent levels of inequality and institutional fragility, these reforms may threaten established bureaucratic structures and informal governance arrangements, leading to resistance and ultimately ineffective implementation. Thus, even when these reforms are legitimate from a regulatory and normative perspective, they frequently prove inoperative in practice because they are misaligned with the expectations and schemas of those responsible for their execution.

During the COVID-19 pandemic, several policies were rapidly enacted to contain the spread of the virus and protect vulnerable populations, particularly in long-term care institutions for older adults. Holahan and Bardakh (2024) note that these measures were both legitimate and widely accepted by society as necessary responses to the public health crisis. However, their implementation exposed a misalignment with the day-to-day operational realities of healthcare professionals in these settings. Doctors, nurses, and caregivers encountered difficulties in applying the restrictions, which—though technically sound—failed to account for the complex needs involved in caring for frail elderly individuals. The exclusion of these professionals from the policy design process further compromised implementation, resulting in resistance, emotional strain, and staff attrition. Indeed, this case illustrates how a policy that is formally valid and publicly endorsed can still fail due to its misalignment with the expectations and working conditions of the public servants responsible for its enforcement (Holahan and Bardakh, 2024).

Borba and De Gomes (2022) investigated how perceptions held by Brazilian federal police officers regarding civilian gun ownership affect the implementation of firearm control policies. Based on a survey of 800 officers across different regions and ranks, the study found that although these officers are legally tasked with enforcing firearm regulations—including the seizure of illegal weapons and the arrest of unauthorized carriers—many believe that gun ownership constitutes an individual right. While they acknowledge that firearms alone do not ensure protection against crime, their responses reveal a dissonance between the normative foundations of firearm control policy and the cultural frameworks of those responsible for its implementation. This incongruence may lead to both covert and overt forms of resistance, ultimately compromising policy effectiveness.

Finally, Scholtes and Schröder-Bäck (2019) analyze policies aimed at installing safety equipment in homes to prevent child injuries—measures promoted by public health authorities and broadly accepted as necessary to reduce domestic accidents. Nevertheless, implementation faces ethical dilemmas among professionals responsible for enforcement, particularly due to a reluctance to impose safety standards that may conflict with the cultural practices and preferences of the families involved. This case illustrates a scenario in which the policy is legitimized both by regulatory authority and public acceptance yet still encounters implementation barriers due to incongruence between its normative content and the value systems of the public servants responsible for its application.

## 6 Discussion

The effectiveness of public policy implementation is a central concern for both scholars and practitioners (Pülzl and Treib, 2007; Robichau and Lynn, 2009), especially in contexts where the regulations that establish such policies face resistance from public agents (DeLeon and DeLeon, 2002). Beyond emphasizing the legitimacy of the law that enacts a given policy, this essay has highlighted the importance of congruence between the policy’s content and the shared cultural schemas of public servants. Drawing on insights from sociological approaches to law (Edelman and Suchman, 1999; Edelman and Talesh, 2011), institutional theory (Dornbusch and Scott, 1975; Scott, 2008; Suchman, 1995), and cultural-cognitive perspectives (D’Andrade, 1992; DiMaggio, 1997; Strauss and Quinn, 1997; Vaisey, 2009; Valentino, 2021; Zerubavel, 1997), we have theorized how both the legal validity of public policies and the propriety attributed by implementing agents play a fundamental role in shaping conformity with the spirit of the law.

As a result, we seek to contribute to the public policy literature—particularly in contexts of top-down implementation, which remain prevalent in modern democracies with large bureaucratic infrastructures (Andrews, 2008; Olsen, 2008)—by systematizing the possible outcomes associated with varying degrees of alignment between legal norms and their implementation. This framework has two key implications for researchers and practitioners.

First, when evaluating the content of a public policy, it is essential to recognize that its effectiveness depends not only on the authority of the entity that enacts it (Koskenniemi, 1997; Wallner, 2008) but also on whether its content is endorsed by society (Rosén, 2017; Rossoni, 2016; Walker, 2004). Scholars aiming to assess the likelihood of successful implementation must therefore consider whether a policy’s content aligns with the expectations, beliefs, and interests of its key stakeholders. Only by identifying potential mismatches can we understand which elements of a policy might undermine its legitimacy (Suchman and Edelman, 1996).

Second, because the success of public policies ultimately depends on the agency of the public servants tasked with implementation (Amirkhanyan et al., 2017), it is crucial to evaluate the extent to which a policy’s content is congruent with the cultural schemas held by these actors. This means recognizing that public servants interpret and rationalize policies (Rossoni et al., 2013), using them as guides for action based on how their elements are represented within the implementers’ prevailing cognitive structures (Abrutyn and Lizardo, 2024; Zaloznaya, 2012).

### 6.1 Methodological implications

From a methodological standpoint, identifying the congruence between policy content and public servants’ schemas requires evaluating key elements of the law in terms of perceived agreement and relevance. This can be achieved using established methods for assessing ideological and cultural congruence (Ajzen and Sexton, 1999; Dressler et al., 2019; Dressler, 2020; Golder and Stramski, 2010; Lu et al., 2024). These include qualitative approaches such as interviews and ethnographic observation, as well as quantitative tools like structured questionnaires and implicit association tests (Cerulo et al., 2021; Miles et al., 2019; Vaisey, 2009).

For the analysis of congruence, linear methods, including regression and its derivatives, may be applied. However, given the complex relationships between validity, propriety, and conformity, it is often necessary to explicitly model moderating effects (Gardner et al., 2017), or to adopt techniques capable of capturing multi-dimensional interactions—such as Response Surface Analysis (Nestler et al., 2019). Since belief systems are relational and composed of interconnected cognitive elements, we also recommend employing methods that reflect this complexity, such as psychometric network analysis (Borsboom, 2022).

Moreover, because schemas represent heterogeneous yet patterned structures of meaning, researchers should use techniques that can uncover diverse shared cognitive patterns across populations. Methods like Relational Class Analysis (Goldberg, 2011) and Correlational Class Analysis (Boutyline, 2017) are particularly useful in this regard. These approaches make it possible to identify subgroups of public servants who share similar cultural schemas (Hunzaker and Valentino, 2019; Rossoni et al., 2021; Willekens and Daenekindt, 2022). In the field of textual analysis, additional tools such as Conceptual Class Analysis (CoCA)—developed by Taylor and Stoltz (2020)—combine word embeddings with correlational class logic to cluster documents based on underlying schema structures. Other approaches based on cosine similarity or more advanced vector space models (Srinivasarao et al., 2022) also offer powerful means of analyzing cultural content.

### 6.2 Concluding remarks

This essay argues that the effectiveness of public policy implementation depends on both the legitimacy of the law that establishes the policy and the congruence of its content with the cultural schemas of public servants. First, we showed that conformity to legal norms is influenced by the law’s validity, understood as the combination of the issuer’s authority and the degree of societal endorsement. Second, we argue that propriety, which emerges from the alignment between a policy’s content and implementers’ shared meaning structures, also shapes conformity. Third, we theorized four possible implementation scenarios based on different combinations of validity and propriety, each associated with a distinct strategic response.

When both validity and propriety are high, the most likely outcome is conformity, accompanied by a response of compromise. In cases where validity is low, but propriety is high, customization is expected, as conflicts over policy goals prompt a strategy of persuasion. Conversely, when validity is high, but propriety is low, customization may still occur, but the conflict lies in the means, and the response tends toward manipulation. Finally, when both validity and propriety are low, implementation is likely to be ceremonial or symbolic, with public servants responding through avoidance or defiance.
